# CT imaging shows specific pancreatic abnormalities in persons with cystic fibrosis related diabetes

**DOI:** 10.1038/s41598-023-37492-4

**Published:** 2023-06-27

**Authors:** Laure Alexandre-Heymann, Marie Puerto, Clémence Martin, Espérie Burnet, Helen Mosnier-Pudar, Pierre-Régis Burgel, Etienne Larger

**Affiliations:** 1 Institut Cochin, INSERM, CNRS, Université Paris Cité, 75014 Paris, France; 2 Service de Pneumologie, National Reference Center for Cystic Fibrosis, ERN-Lung CF Network, AP-HP, Hôpital Cochin, 75014 Paris, France; 3 Service de Diabétologie, AP-HP, Hôpital Cochin, 123 Boulevard de Port-Royal, 75014 Paris, France

**Keywords:** Anatomy, Endocrinology, Medical research, Signs and symptoms

## Abstract

Cystic fibrosis related diabetes (CFRD) is observed in 20–50% of adults with cystic fibrosis (CF). Pancreas abnormalities on imaging, e.g. pancreas lipomatosis, are frequent in subjects with CF. We hypothesized that specific abnormalities may characterize patients with CFRD. We performed a retrospective study comparing the computed tomography (CT) of participants with CF with or without diabetes (“CFRD” and “CF control” groups). We classified the pancreas on imaging according to 3 categories: normal, partial lipomatosis and complete lipomatosis of the pancreas. We also assessed the presence or absence of pancreatic calcifications. Forty-one CFRD and 53 CF control participants were included. Only 2% of the patients with CFRD had a normal pancreas, as compared with 30% of the participants from the CF control group (*p* = 0.0016). Lipomatosis was more frequent in subjects with CFRD and was related to exocrine pancreatic insufficiency (EPI) and to severe *CFTR* mutations (classes I to III). Nine participants with diabetes (22%) presented with pancreatic calcifications, versus none of the control participants (*p* = 0.0003). In conclusion, pancreas imaging was almost always abnormal in subjects with CFRD, while it was normal in a third of the CF control subjects. Pancreatic calcifications were specific of subjects with CFRD.

Cystic fibrosis (CF) affects about one out of every 3000 newborns of Northern European ancestry. It is a multisystem disease that can affect the respiratory tract, the gastrointestinal tract, the gonads, the liver, and the pancreas. Disorders of the endocrine pancreas can also occur: one of the common complications of CF is cystic fibrosis related diabetes (CFRD), a specific form of diabetes. CFRD is found in 2% of children, 7–20% of adolescents, and 20–50% of adults with CF^1–3^ and is more often seen in women than in men. CFRD can lead to classic diabetes microvascular complications such as retinopathy or nephropathy^4^ but is also associated with overall worse clinical status and worse pulmonary function^1,5,6^. Until recently, the mortality rate was significantly higher in persons with CFRD than in persons without diabetes^7^ but this gap has narrowed in recent years, which is likely due to improved screening and treatment of CFRD^2,8^.

Lesions of the exocrine pancreas are one of the hallmarks of CF: persons with CF are known to present with structural abnormalities of the exocrine pancreas such as cysts, fatty replacement, atrophy, or fibrosis of the exocrine gland^9^. These abnormalities can be detected on abdominal ultrasounds (AUS), computed tomography (CT) or magnetic resonance imaging^9–12^. Endocrine lesions of the pancreas can also be found in children with CF, even before lipomatosis has developed, and the severity of these endocrine lesions does not seem to correlate with the extent of the exocrine lesions^13^.

More generally, outside the field of CF, the association between endocrine and exocrine pancreatic anomalies, including on CT imaging, has been a subject of growing interest in the last decade^14–16^: abnormalities of the exocrine pancreas have been described in patients with type 1 (T1D) or type 2 diabetes (T2D) since the beginning of the twentieth century^17,18^. The pancreas volume is lower in persons with T1D than in the general population, and acinar atrophy, pancreatic fibrosis or adipose infiltration are common findings in subjects with diabetes. Therefore, we hypothesized that in persons with CF, pancreatic imaging may also differ between persons with and without diabetes.

However, only a few studies have looked for an association between CFRD and the morphology of the pancreas: Iannucci et al.^19^ showed that even though all persons with CF had fatty replacement of the exocrine pancreas at autopsy, persons without CFRD still had some healthy exocrine tissue around their Langerhans islets, which was not found in persons with CFRD. Other studies^20–22^, conducted on small series of participants, showed that fatty replacement and atrophy are very frequent observations in persons with CF. These studies found no difference between persons with and without CFRD.

Hence, the aim of our study was to compare the pancreatic findings on CT abdominal imaging between participants with CF with or without CFRD. Our primary outcome was to compare the prevalence of pancreas lipomatosis, as it is the most common exocrine pancreas abnormality in CF. The secondary outcome was to compare the prevalence of pancreas calcifications, which have been shown to be associated with diabetes in chronic pancreatitis from other causes^23^.

## Methods

### Subjects

We performed a retrospective study comparing participants with CF, with or without CFRD. It was conducted at the adult National Referral Center for Cystic Fibrosis (Cochin Hospital, Paris), which followed over 500 adults with CF in 2019.

Participants were eligible if they had CF, were older than 18 years, and had undergone abdominal CT between 2008 and 2016 in Cochin hospital, Paris. Using the data available from previous studies^20,21^, we estimated that we needed at least 35 participants in each group in order to detect a 25% difference between the groups (anticipated prevalence of partial or complete lipomatosis of the pancreas in participants without CFRD: 70%^22^, alpha risk: 0.05, study power: 80%).

**Figure 1:**
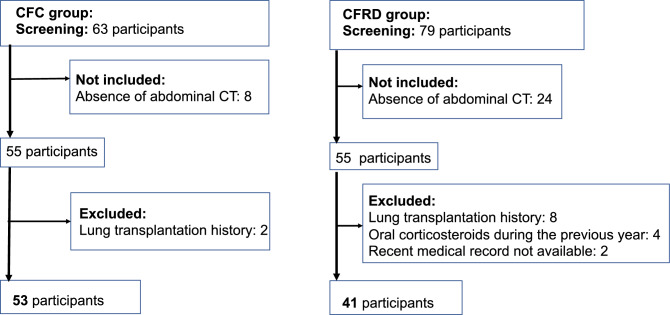
Flowchart.

The participants were randomly selected from the large database established by the Reference Center for Cystic Fibrosis and were included only if they had undergone an abdominal CT between 2008 and 2016, until 55 participants with CFRD (diagnosed before their last available abdominal imaging was performed) and 55 participants with CF but without diabetes were included, in order to retain enough participants after exclusion (Fig. 1).

All clinical and biological data were extracted from the participants’ electronic medical records.

The diagnosis of diabetes was based on the following criteria: fasting glycemia > 7 mmol/L on two different samplings AND/OR > 11 mmol/L, 2 h after a 75 g oral glucose tolerance test (OGTT), AND/OR an already ongoing treatment for diabetes (oral treatment or insulin) at first appointment. Participants diagnosed with diabetes were classified as participants with diabetes (CFRD group), while the others were classified as participants without diabetes (CF control group).

Participants were excluded if they met with one of the following exclusion criteria: a history of lung transplantation; a history of treatment with oral corticosteroids for any reason in the year prior to the abdominal imaging; or the lack of recent medical records in the hospital database (maximum two years before the abdominal imaging). Two participants from the CF control group were excluded because they had a history of lung transplantation. In the CFRD group, 8 participants were excluded because they had a history of lung transplantation, 4 because they had been treated by oral corticosteroids in the previous year, and 2 because a recent medical record was not available.

CF control group participants’ blood test results from 2017 to 2019 were also reviewed, to find out if participants from the CF control group developed CFRD after the study endpoint.

### Materials and methods

#### Abdominal imaging

**Figure 2:**
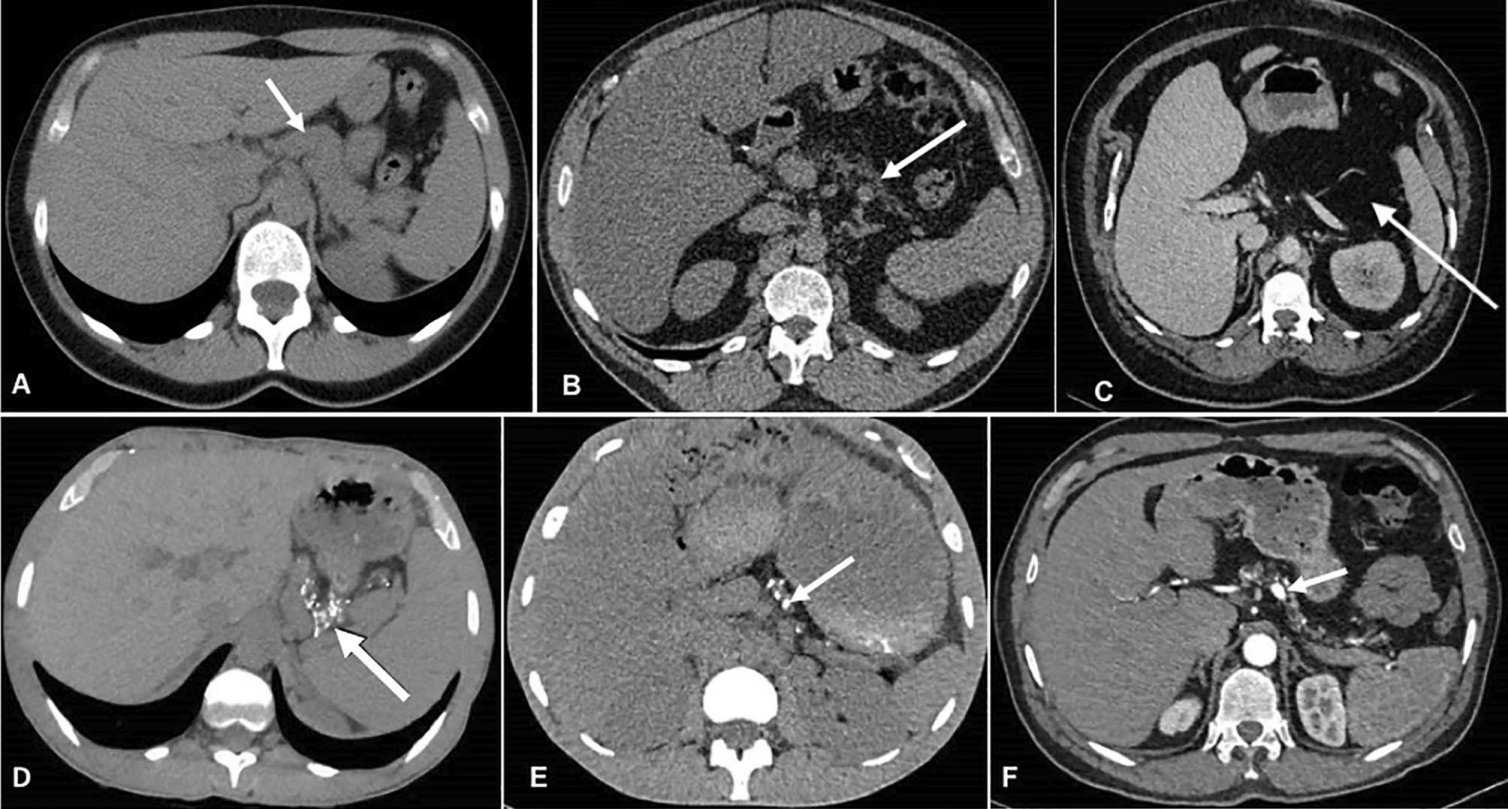
Computed tomography without contrast Top row: Different degrees of pancreas lipomatosis. (**a**) Normal pancreas (**b**) Partial lipomatosis of the pancreas. (**c**) Complete lipomatosis of the pancreas (white arrow shows the visually empty pancreatic area.) Bottom row (**d–f**): three examples of pancreatic calcifications (arrows). Of note, all images of bottom row show partial lipomatosis of the pancreas.

All the CT scans were assessed by two investigators (LAH and EL), who were blinded to the diabetes status of the participants. The pancreas on CT were classified according to the following 3 categories:

A: normal pancreas (Fig. 2A); B: partial lipomatosis: pancreas still visible, although hypotrophic (Fig. 2B); C: complete lipomatosis: no visible pancreas on the CT images (Fig. 2C).

In addition, the presence of pancreatic calcifications was assessed (Fig. 2D–F).

When available, abdominal ultrasounds (AUS) results were also assessed. They could not be reviewed as they were captured in real-time. Therefore, written reports were used to classify the results according to the following categories: A: normal pancreas; B: partial lipomatosis of the pancreas (pancreas described as hypotrophic and/or hyperechoic); C: complete lipomatosis of the pancreas (complete lipomatosis of the pancreas specifically noted on the report); D: pancreatic cyst(s) (presence of pancreatic cyst(s) in a pancreas otherwise described as normal); E: pancreas “not visualized” (which could either mean complete lipomatosis of the pancreas or difficult conditions of imaging); F: uninterpretable results (described as uninterpretable in the report, usually because of intestinal or air interpositions).

#### Exocrine pancreatic insufficiency

Exocrine pancreatic insufficiency (EPI) was assessed and deemed present if fecal elastase-1 was < 100 µg/g AND/OR if the patient was already taking pancreatic enzyme replacement therapy.

#### CFTR mutations

Cystic Fibrosis Transmembrane conductance Regulator *(CFTR)* mutations were classified according to The Clinical and Functional TRanslation of CFTR (CFTR2); available at http://cftr2.org, as severe (classes I to III) or moderate (classes IV–VI)^24,25^. Participants were further classified as having 0, 1 or 2 severe *CFTR* mutations.

### Statistical analysis

Statistics were performed using Graphpad Prism 5 (GraphPad Software, Inc., San Diego, CA). Data are presented as mean and standard deviation (SD) for normally distributed variables, and as median and interquartile range (IQR) for variables with a non-normal distribution. For continuous variables, unpaired t-test was used when 2 groups of normally distributed variables were compared, and one-way ANOVA was used when more than 2 groups were compared. Mann–Whitney test was used when 2 groups of variables with a non-normal distribution were compared, and Kruskall-Wallis test was used when more than 2 groups were compared. For categorical variables, Fisher’s exact test was used when 2 groups were compared and Chi-square was used when more than 2 groups were compared. A *p*-value < 0.05 was considered statistically significant.

We performed several subgroup analyses: we analysed a subgroup of CF control and CFRD participants matched on age so as to avoid a possible age bias; we compared the subgroups of CF control and CFRD participants who specifically presented with EPI; we compared the subgroups of CF control and CFRD participants who specifically presented with two severe CFTR mutations in order to take disease severity into account; finally, among participants with CFRD, we compared participants with and without pancreatic calcifications. Moreover, we also performed an overall analysis of the total population stratified by CT findings.

When data was missing, the number of analysable participants for the concerned variable was reported in the corresponding table.

### Statement of ethical approval

This was a retrospective study, using existing clinical data only. According to the ethics regulation and French law, all the included participants were informed of the study. The informed consent was waived by the ethics committee that has approved the study according to French law due to the retrospective nature of the study. All the medical data were anonymized. All methods were performed in accordance with the relevant guidelines and regulations. The database was registered with the Commission Nationale Informatique et Libertés (CNIL) registration no.: 2203351v0. The study was approved by the Institutional Review Board of The French learned Society for Respiratory Medicine (Société de Pneumologie de Langue Française) (#CEPRO 2020-052**).**

## Results

Forty-one participants with CFRD and 53 participants with CF but without CFRD were analyzed. The median age was 32 years, 52% of the participants were men. The participants with CFRD were older than the participants from the CF control group (33 vs. 30 years, *p* = 0.05), presented more often with exocrine pancreatic insufficiency and with 2 severe *CFTR* mutations (Supplementary Table 1) than the participants from the CF control group (*p* < 0.0001 and *p* = 0.003, respectively) (Table 1).

**Table 1:** Clinical characteristics of the participants.

	CF control participants N = 53	CFRD participants N = 41	*p*
Age *years, median (IQR)*	30 (25–37)	33 (30–41)	0.05
Men *n (%)*	28 (53)	21 (51)	1
BMI^a^ *kg/m*^2^*: mean (SD)*	21.7 (2.9)	21.1 (3.1)	0.35
Exocrine pancreatic insufficiency *n (%)*	35 (66)	41 (100)	< 0.0001
Two severe *CFTR* mutations *n (%)*	38 (72)	39 (95)	0.003
Blood tests (until 2016)			
Highest known HbA_1c_^a^*%: median (IQR)*	5.7 (5.4–5.9)	7.5 (6.9–8.5)	< 0.0001
Highest known HbA_1c_^a^*mmol/mol: median (IQR)*	39 (36–41)	58 (52–68)	
Highest known fasting glycemia^b^ *mmol/L: median (IQR)*	5.1 (4.6–5.4)	7.7 (6.2–10.6)	< 0.0001

*CF control participants* participants with cystic fibrosis but without diabetes, *CFRD participants* cystic fibrosis related diabetes, *IQR* interquartile range, *BMI* body mass index, *SD* standard deviation.

^a^Available in 45 control and 38 diabetes participants.

^b^Available in 49 control and 39 diabetes participants.

The age-matched subgroup comprised 32 participants in each group (CF control and CFRD groups), the overall median age was 32.5 years. Similarly to the main analysis, participants with CFRD in this subgroup presented more often with EPI and with 2 severe *CFTR* mutations than the participants from the CF control group (100% vs. 63%, *p* = 0.0001; and 97% vs. 72%, *p* = 0.01).

In 90 patients, at least one AUS had been performed. Nine AUS were described as uninterpretable because of air or intestinal interpositions, and AUS and CT results were discordant in 24 patients. Thus, among the patients with both AUS and CT available, 37% of the AUS results were either uninterpretable or discordant with the CT results (Supplementary Table 1). It must also be noted that, when the patients had multiple abdominal AUS, the results were very variable from one assessment to the other (data not shown). Therefore, only the results of the CT scans were used for the rest of this study.

Eighty-three percent of the abdominal CT scans were performed for cystic fibrosis regular follow-up, 6% for abdominal pain, 5% for pre-transplant evaluation, and 5% for other reasons (namely fever or suspicion for acute pancreatitis).

### Comparison between participants with and without CFRD

**Figure 3:**
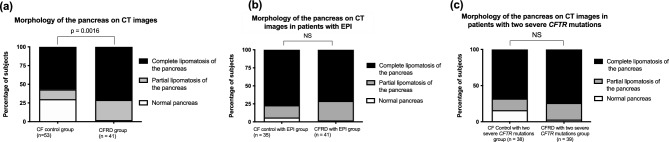
Morphology of the pancreas on CT images in participants with CF without CFRD (CF control group) or with CFRD (CFRD group) (**a**) Comparison between the participants from the CF control and the CFRD group (**b**) Comparison between the participants from the CF control subgroup who presented with EPI (control with EPI group) and the participants with CFRD. (**c**) Comparison between the participants from the CF control and CFRD subgroups who presented with 2 severe mutations of *CFTR*. EPI: exocrine pancreatic insufficiency. Black: complete lipomatosis of the pancreas. Grey: partial lipomatosis of the pancreas. White: normal pancreas.

Two percent of the patients with CFRD presented with a normal pancreas on imaging, as compared with 30% of the patients in the CF control group (*p* = 0.0016). Fifty-seven percent of the participants from the CF control group presented with complete lipomatosis of the pancreas, versus 71% of the participants from the CFRD group (Fig. 3a). Similarly, in the age-matched subgroup, 59% of the participants from the CF control group presented with complete pancreatic lipomatosis, versus 72% of the participants from the CFRD group (*p* = 0.002).

Since EPI is an important risk factor for the development of CFRD^26^, we also compared the participants from the CFRD and CF control groups who specifically presented with EPI. Participants with EPI from the CF control group and from the CFRD group presented with a similar rate of complete pancreatic lipomatosis (27 (77%) vs. 29 (71%) participants, NS) (Fig. 3b). Conversely, 78% of the CF control participants without EPI had a normal pancreas on CT scan.

Since genotype severity is also related to the presence of CFRD^27^, we compared the CFRD and CF control participants who specifically presented with two severe *CFTR* mutations. Twenty-nine out of 39 (74%) participants with CFRD and two severe mutations presented with complete lipomatosis of the pancreas, versus 26 out of 38 (68%) CF control participants with two severe mutations, and the difference between the groups was not statistically significant (Fig. 3c). Of note, 67% of the CF control participants who did not present with two severe *CFTR* mutations had a normal pancreas on CT scan.

Unexpectedly, 9 participants (22%) with CFRD presented with pancreatic calcifications, versus none of the CF control participants (p = 0.0003). None of these 9 participants presented with a history of acute pancreatitis. The pattern of calcifications was heterogeneous: some participants presented with small and numerous calcifications scattered among the remaining pancreas (Fig. 2d), while others presented with only one or several voluminous calcifications (Fig. 2f). Calcifications were often associated with small parts of remaining pancreatic tissue visible on CT scan. Thus, in the CFRD group, the percentage of participants with complete pancreatic lipomatosis was lower in the subgroup with pancreatic calcifications than in the subgroup without pancreatic calcifications (*p* = 0.01). No other clinical difference was found between those two subgroups (Table 2).

**Table 2:** Clinical characteristics of the participants with CFRD, with or without calcifications on CT images.

	No calcification N = 32	Calcifications N = 9	*p*
Age *years: median (IQR)*	34 (31–42)	30 (27–38)	0.3
Men *n (%)*	16 (50)	5 (56)	1
BMI^a^ *kg/m*^*2*^*: mean (SD)*	21.2 (3.1)	21.0 (3.3)	0.9
Two severe *CFTR* mutations n* (%)*	30 (94)	9 (100)	1
Age at diagnosis of diabetes *years: median (IQR)*	23 (18–30)	21 (15–25)	0.24
Duration of diabetes at time of abdominal imaging *years: median (IQR)*	12 (8–14)	12 (1.5–20.5)	0.9
Highest known HbA_1_c^a^ *%: median (IQR)*	7.5 (6.8–8.7)	7.4 (7–8.1)	0.9
Highest known HbA1c *mmol/mol: median (IQR)*	58 (51–72)	57 (53–65)	
Currently treated with insulin *n (%)*	29 (91)	8 (89)	1
Complete pancreatic lipomatosis on CT *n (%)*	26 (81)	3 (33)	0.01

*IQR* interquartile range, *BMI* body mass index, *SD* standard deviation.

^a^Available in 29 participants without calcifications and 9 with calcifications.

Finally, 2 men and 1 woman from the CF control group, *i.e.* participants without diabetes until 2016, were diagnosed with diabetes between 2016 and 2019. None of them were glucose intolerant before 2016. They were respectively 56, 32, and 24 years old at diagnosis of diabetes. All of them had EPI. The oldest patient had only one severe *CFTR* mutation, the other participants had 2 severe *CFTR* mutations. Two participants had complete lipomatosis of the pancreas on the CT scans performed respectively 2 years and 1 year before diabetes was diagnosed. However, the 32-year-old patient, who presented with fasting hyperglycemia but a normal HbA_1c,_ had a normal abdominal CT 2 years before being diagnosed with diabetes.

### Comparison between participants with and without complete pancreatic lipomatosis on CT scan

Irrespective of diabetes or control group, 17 participants had a normal pancreas on CT, 18 presented with partial pancreatic lipomatosis and 59 presented with complete pancreatic lipomatosis. Age, gender ratio and BMI were similar between the 3 groups. Only 6% of the participants with a normal CT presented with CFRD, versus 61% and 52% of the participants with partial and complete pancreatic lipomatosis, respectively (*p* = 0.0016).

EPI was observed in only 29% of the participants with a normal pancreas on CT images versus in 94% and 95% of the participants with partial and complete pancreatic lipomatosis, respectively (*p* < 0.0001).

Nine percent of the patients with two severe *CFTR* mutations had a normal pancreas on CT scan, versus 63% of the patients with zero or one severe *CFTR* mutation. Seventy-two percent of the patients with two severe *CFTR* mutations had complete pancreatic lipomatosis, versus 19% of the patients with zero or one severe mutation (*p* < 0.0001).

Interestingly, 2% of the participants with CFRD and 17% of the CF control participants had a history of acute pancreatitis. Among the participants with a normal pancreas on CT scan, 29% had a history of acute pancreatitis, versus 16% in those with partial pancreatic lipomatosis and only 3% of the patients with complete pancreatic lipomatosis (*p* = 0.0059).

## Discussion

The relationships between exocrine and endocrine pancreas are a subject of growing interest. Indeed, many studies have shown structural and functional exocrine pancreatic abnormalities in type 1 and type 2 diabetes^15,16,28^, and diabetes is a complication of many pancreatic diseases (chronic pancreatitis, pancreatic cancer, CF)^23^. Fatty degeneration of the pancreas is a quite common anomaly in type 2 diabetes^29,30^. Hence, we wanted to determine if the pancreas of patients with CFRD showed specific characteristics as compared to patients with CF but without diabetes. To our knowledge, this is the first large study comparing the CT characteristics of the pancreas of people with CF with or without CFRD.

We found several differences between participants with and without CFRD. Indeed, the prevalence of complete lipomatosis of the pancreas was higher in participants with CFRD related to a more severe genotype in this group and a higher prevalence of EPI. We also showed the presence of pancreatic calcifications in about one-quarter of participants with CFRD, versus in none of the participants from the CF control group.

In order to evaluate pancreas morphology, we had first planned to use both CT and AUS imaging, but conventional AUS results were not reproducible and were often discordant with the CT results. This question had already been raised in studies that compared conventional AUS and CT scan for the evaluation of pancreatic adenocarcinoma, with conflicting results^31,32^. Therefore, conventional AUS should not be chosen to evaluate the state of the pancreas in persons with CF.

The prevalence of complete lipomatosis of the pancreas in the present study was comparable to that reported by Sequeiros et al., who used MRI and involved only 13 participants with CFRD and 16 participants with CF without CFRD. In that study, the prevalence of complete lipomatosis of the pancreas was 77% in participants with CFRD and 69% in participants without CFRD (vs. 71% and 57% respectively in the present study). Thus, participants with CFRD presented more often with complete lipomatosis of the exocrine pancreas than participants from the CF control group. This was explained by the overall more severe genotype in the CFRD than in the CF control group: the prevalence of CFRD is known to be higher in patients with two severe *CFTR* mutations^33^. Moreover, the prevalence of CFRD is also known to be higher in patients with EPI^33^, and EPI is associated with a higher prevalence of partial or complete pancreatic lipomatosis^21^. Hence, when we compared participants with two severe *CFTR* mutations with or without CFRD, the prevalence of pancreatic lipomatosis did not differ between groups. Similarly, the prevalence of complete pancreatic lipomatosis was not different between participants with EPI from the diabetes and CF control groups. Complete pancreatic lipomatosis could thus hardly be used as a distinctive criterion for persons with CFRD, all the more since one of the participants from the CF control group that developed diabetes afterwards had a normal abdominal CT scan 3 years before being diagnosed with diabetes.

However, it is interesting to note that the patients with a normal pancreas on CT scan were at a much higher risk of acute pancreatitis than the ones with complete pancreatic lipomatosis. This is also probably explained by the association between EPI and complete pancreatic lipomatosis, since studies have shown that pancreatic sufficient subjects are more prone to acute pancreatitis than subjects with EPI^21,34,35^.

Another noteworthy result of this study is that pancreatic calcifications were found in almost one-fourth of participants with CFRD and were not seen in participants without CFRD. Participants with pancreatic calcifications presented more often with remaining zones of exocrine pancreatic tissue. No clinical difference was observed between participants with and without calcifications. To our knowledge, the association between CFRD and pancreatic calcifications has been hinted at since 1963^36^ but has never been evaluated in large series of adult persons with CFRD. A study including 60 participants with CF older than 5 years old showed a prevalence of pancreatic calcifications of 8%. Eighty percent of the participants with pancreatic calcifications had at least glucose intolerance^37^. Interestingly, in non CF persons with chronic pancreatitis, people with pancreatic calcifications are at higher risk of diabetes than people without calcifications^23^.

Therefore, we reckon that abdominal imaging should not be performed in subjects with CF in order to screen for CFRD because sensitivity and specificity would be too low; however, physicians should look more closely for CFRD in subjects with pancreas calcifications, which can be found on abdominal slices of routinely performed thoracic CT.

It must be noted that CFTR modulators were not widely available in France during the period of inclusion. Thus, the included participants did not receive this class of treatment at the time. CFTR modulators seem to improve exocrine function in children^38^. It will therefore be interesting to study if they will decrease both exocrine and endocrine failure, and to follow pancreas CT imaging in subjects treated with CFTR modulators from a young age.

Our study bears several limitations. Participants with and without CFRD were not matched for gender, age or BMI. This could induce a bias in the interpretation of results. However, the gender-ratio and BMI were similar between the two groups, and results in the age-matched subgroup were similar to the ones in the overall study.

Moreover, it was a retrospective study and the number of participants with pancreatic calcifications was small. Therefore, these observations need to be confirmed in larger prospective series of adult persons with CF.

## Conclusion

In this large study comparing the pancreas of participants with CF with or without CFRD, we showed that complete pancreatic lipomatosis was more often seen in participants with CFRD than in participants without, associated with an overall more severe genotype in the CFRD group. A more distinctive finding was the presence of pancreatic calcifications, found in almost one in four participants with CFRD and not in participants without CFRD. Pancreatic lipomatosis cannot be used to differentiate between subjects with or without CFRD since it is a very common occurrence in both groups, but physicians should look more closely for diabetes in subjects with pancreas calcifications on abdominal imaging.

## Supplementary Information

Supplementary Tables.

## Data Availability

The datasets generated during and/or analysed during the current study are available from the corresponding author on reasonable request.
