# Supplementary material for: CT imaging shows specific pancreatic abnormalities in persons with cystic fibrosis related diabetes
Source: Sci Rep. 2023 Jun 27;13:10433. doi: 10.1038/s41598-023-37492-4 (PMC10300009; doi:10.1038/s41598-023-37492-4)
Supplement: Supplementary file 1 — Supplementary Tables. [file 41598_2023_37492_MOESM1_ESM.docx]

Supplementary material

| ***CFTR* mutations (CF control participants)** | **Pancreas lipomatosis** |
| --- | --- |
| delta F508 / R334W | Normal |
| delta F508 / L165S | Normal |
| delta F508 / W1282X | Normal |
| delta F508 / R1070W | Normal |
| delta F508 / 3849+10kb C->T | Normal |
| G85E/R334W | Normal |
| delta F508 / S945L | Normal |
| G85E/R764X | Normal |
| delta F508 / delta F508 | Normal |
| delta F508 / 3849+10kb C->T | Normal |
| delta F508 /S1159F | Normal |
| R334W/R792X | Normal |
| delta F508 / L206W | Normal |
| F508del / M1101 K | Normal |
| p.R117C/p.1200-1110_1113delTAAG | Normal |
| G551D/R347H | Normal |
| delta F508 / Y122X | Partial lipomatosis |
| R553X/E92X | Partial lipomatosis |
| delta F508 / delta F508 | Partial lipomatosis |
| delta F508 / delta F508 | Partial lipomatosis |
| delta F508/R334W | Partial lipomatosis |
| delta F508 / delta F508 | Partial lipomatosis |
| L558S(exon 11)/2183 AA->G | Partial lipomatosis |
| delta F508 / delta F508 | Complete lipomatosis |
| N1303K/1898+5 G->A | Complete lipomatosis |
| delta F508 / p.R75X-p.R668C | Complete lipomatosis |
| T1086I/E292K | Complete lipomatosis |
| delta F508 / delta F508 | Complete lipomatosis |
| delta F508 / delta F508 | Complete lipomatosis |
| delta F508 / delta F508 | Complete lipomatosis |
| delta F508 / Y1092X | Complete lipomatosis |
| G551D/S492F | Complete lipomatosis |
| delta F508 / delta F508 | Complete lipomatosis |
| delta F508 / delta F508 | Complete lipomatosis |
| delta F508 / delta F508 | Complete lipomatosis |
| delta F508 / 1249 5AG | Complete lipomatosis |
| delta F508 1717 1GA | Complete lipomatosis |
| delta F508 / delta F508 | Complete lipomatosis |
| delta F508 / delta F508 | Complete lipomatosis |
| delta F508 / delta F508 | Complete lipomatosis |
| delta F508 / delta F508 | Complete lipomatosis |
| delta F508 / delta F508 | Complete lipomatosis |
| deltaF508 / G542X | Complete lipomatosis |
| deltaF508 / 1717-1G>A | Complete lipomatosis |
| delta F508 / delta F508 | Complete lipomatosis |
| delta F508 / delta F508 | Complete lipomatosis |
| del exons 17-18/del exons 17-18 | Complete lipomatosis |
| R553X / 3154 del G | Complete lipomatosis |
| ΔF 508/R553X | Complete lipomatosis |
| delta F508 / delta F508 | Complete lipomatosis |
| delta F508 / delta F508 | Complete lipomatosis |
| F 508del / M1101K | Complete lipomatosis |
| delta F508 / delta F508 | Complete lipomatosis |

| ***CFTR* mutations (CFRD participants)** | **Pancreas lipomatosis** | **Pancreas calcifications** |
| --- | --- | --- |
| delta F508 / delta F508 | Normal | No |
| delta F508 / 3849+10kbC -> T | Partial lipomatosis | No |
| delta F508 / delta F508 | Partial lipomatosis | **Yes** |
| delta F508 / delta F508 | Partial lipomatosis | **Yes** |
| Homozygous *CFTR* large deletion | Partial lipomatosis | **Yes** |
| 4016insT/4016insT | Partial lipomatosis | **Yes** |
| G551D/ D1152H | Partial lipomatosis | No |
| deltaF508/1066L | Partial lipomatosis | No |
| deltaF508/Q493X | Partial lipomatosis | No |
| F508del / W1282X | Partial lipomatosis | **Yes** |
| delta F508 / delta F508 | Partial lipomatosis | **Yes** |
| dele 9_7/3040+1G<A | Partial lipomatosis | No |
| N1303K / N1303K | Complete lipomatosis | No |
| delta F508 / delta F508 | Complete lipomatosis | No |
| delta F508 / delta F508 | Complete lipomatosis | No |
| deltaF508/2183AA>G | Complete lipomatosis | No |
| Y122X / Y122X | Complete lipomatosis | No |
| delta F508 / delta F508 | Complete lipomatosis | No |
| delta F508 / exon 10 1677delTA | Complete lipomatosis | **Yes** |
| delta F508 / delta F508 | Complete lipomatosis | No |
| delta F508/N1303K | Complete lipomatosis | No |
| delta F508 / 2622+1G>A | Complete lipomatosis | No |
| delta F508 / delta F508 | Complete lipomatosis | No |
| W1282X / W1282X | Complete lipomatosis | **Yes** |
| delta F508 / delta F508 | Complete lipomatosis | No |
| delta F508 / delta F508 | Complete lipomatosis | No |
| delta F508/I507 del | Complete lipomatosis | No |
| N1303K/E1104 | Complete lipomatosis | No |
| delta F508 / delta F508 | Complete lipomatosis | No |
| delta F508 / delta F508 | Complete lipomatosis | No |
| Delta F508 /2894insAG | Complete lipomatosis | No |
| delta F508 / delta F508 | Complete lipomatosis | No |
| ΔF508/W1282X | Complete lipomatosis | No |
| delta F508/del 17a-17b-18 | Complete lipomatosis | No |
| delta F508 / delta F508 | Complete lipomatosis | No |
| delta F508 / delta F508 | Complete lipomatosis | No |
| G542X/ L1303 | Complete lipomatosis | No |
| 1677delTA/ V754M-CFTRdele3-10+14b-16 | Complete lipomatosis | **Yes** |
| delta F508 / delta F508 | Complete lipomatosis | No |
| delta F508 / delta F508 | Complete lipomatosis | No |
| delta F508 / delta F508 | Complete lipomatosis | No |

**Supplementary table 1**

***CFTR* mutations in participants with CF without or with CFRD, and associated pancreas lesions on CT.**

| **Computed Tomography (CT)**  **Ultrasounds** | **Normal** | **Partial or complete lipomatosis of the pancreas** |
| --- | --- | --- |
| **Normal** | **14** | 17 |
| **Partial or complete lipomatosis of the pancreas, or « pancreas not visualized »** | 2 | **43** |
| **Pancreatic cyst** | 0 | 5 |
| **Uninterpretable** | 1 | 8 |
| **No abdominal ultrasounds available** | 0 | 4 |

**Supplementary Table 2:** Concordance between ultrasounds and computed tomography.

AUS written reports were used to classify the results according to the following categories:

A: normal pancreas; B: partial lipomatosis of the pancreas (pancreas described as hypotrophic and/or hyperechoic); C: complete lipomatosis of the pancreas (complete lipomatosis of the pancreas specifically noted on the report); D: pancreatic cyst(s) (presence of pancreatic cyst(s) in a pancreas otherwise described as normal); E: pancreas “not visualized” (which could either mean complete lipomatosis of the pancreas or difficult conditions of imaging); F: uninterpretable results (described as uninterpretable in the report, usually because of intestinal or air interpositions).
